# Adipose Tissue-Derived Products May Present Inflammatory Properties That Affect Chondrocytes and Synoviocytes from Patients with Knee Osteoarthritis

**DOI:** 10.3390/ijms241512401

**Published:** 2023-08-03

**Authors:** Carola Cavallo, Angelo Boffa, Manuela Salerno, Giulia Merli, Brunella Grigolo, Giuseppe Filardo

**Affiliations:** 1 Laboratorio RAMSES, IRCCS Istituto Ortopedico Rizzoli, 40136 Bologna, Italy; carola.cavallo@ior.it (C.C.); brunella.grigolo@ior.it (B.G.); 2 Clinica Ortopedica e Traumatologica 2, IRCCS Istituto Ortopedico Rizzoli, 40136 Bologna, Italy; angelo.boffa@ior.it; 3 Applied and Translational Research (ATR) Center, IRCCS Istituto Ortopedico Rizzoli, 40136 Bologna, Italy; giulia.merli@ior.it (G.M.); ortho@gfilardo.com (G.F.)

**Keywords:** osteoarthritis, OA, adipose tissue, MF-AT, BMI, inflammation

## Abstract

Adipose tissue-derived cell-based injectable therapies have been demonstrated to have disease-modifying effects on joint tissues in preclinical studies on animal osteoarthritis (OA) models, but clinical results are heterogeneous and not always satisfactory. The aim of this study was to investigate the influence of adipose tissue properties on the therapeutic effects of the adipose-derived product in an in vitro OA setting. Micro-fragmented adipose tissue (MF-AT) samples were obtained from 21 OA patients (mean age 51.7 ± 11.8 years, mean BMI 25.7 ± 4.1 kg/m^2^). The analysis of the MF-AT supernatant was performed to analyze the release of inflammatory factors. The effects of MF-AT inflammatory factors were investigated on chondrocytes and synoviocytes gene expression levels. Patients’ characteristics were analyzed to explore their influence on MF-AT inflammatory molecules and on the MF-AT effects on the gene expression of chondrocytes and synoviocytes. The study results demonstrated that adipose tissue-derived products may present inflammatory properties that influence the therapeutic potential for OA treatment, with products with a higher pro-inflammatory profile stimulating a higher expression of genes related to a more inflamed and catabolic phenotype. A higher pro-inflammatory cytokine pattern and a higher pro-inflammatory effect were found in adipose tissue-derived products obtained from OA patients with higher BMI.

## 1. Introduction

Mesenchymal stromal cells (MSCs) have been proposed as ideal candidates for osteoarthritis (OA) treatment thanks to their structural contribution to tissue repair, and even more so, their immunomodulatory, anti-inflammatory, and anabolic paracrine effects [1,2,3,4]. MSCs can be easily isolated from various tissues including bone marrow, peripheral blood, synovial membranes, tendons, skeletal muscle, periosteum, and umbilical cord blood [5]. More recently, adipose tissue is becoming the preferred MSC source for OA management due to its abundance, ease of harvest, and the high number of MSCs and pericytes (precursors to MSCs) that can be obtained compared to the other sources [6,7,8].

Adipose tissue-derived cell-based injectable therapies have been demonstrated to exert disease-modifying effects on joint tissues in preclinical studies on animal OA models [9]. In particular, they showed an ability to structurally improve both cartilage and synovial membrane properties, as well as a decrease in serum and synovial fluid levels of both inflammatory and cartilage degradation markers [9]. These products were recently applied in a clinical setting for the treatment of OA joints, resulting in a safe profile and providing pain reduction and joint functional improvement in several studies [10,11,12,13]. However, clinical evidence on this cell therapy is still limited, with only a few high-level trials published and a high heterogeneity in the reported results with often not completely satisfactory clinical outcomes [14,15,16]. The heterogeneity in the clinical results could be related to the possible high variability of the injected adipose-derived products, which could be different based not only on the different processing techniques, but also on the different characteristics of the OA patients from which the product was obtained. For example, it is known that metabolic syndrome, a very common comorbidity in OA patients, is associated with chronic low-grade inflammation of the adipose tissue [17]. This inflammation component could alter the quality of the adipose tissue and, consequently, the therapeutic potential of the adipose-derived injectable product. It would, therefore, be crucial to understand if adipose-derived products with different inflammatory characteristics have different therapeutic potential for the treatment of OA joints, and which characteristics of the patients could influence the quality of the adipose tissue.

The primary aim of this study was to investigate the influence of adipose tissue properties on the therapeutic effects of the adipose-derived product in an OA setting. The secondary outcome was to evaluate the influence of the patients’ characteristics on the quality of the obtained adipose tissue-derived products and their effects on cells targeted for OA treatment.

## 2. Results

### 2.1. Analysis of the MF-AT Inflammatory Factors

In total, micro-fragmented adipose tissue (MF-AT) samples were obtained from 21 patients undergoing the preparation of an adipose-derived product for OA treatment. Out of these, supernatants derived from MF-AT were collected from 16 patients (7 women and 9 men, mean age of 52.9 ± 11.1 years and a mean Body Mass Index (BMI) of 25.9 ± 4.4 kg/m^2^), since for the remaining 5 patients there was not enough product to perform all the analyses. Out of the 46 inflammatory factors analyzed, 16 were detectable in the MF-AT supernatants from at least 50% of patients and were considered for the purpose of this study: Basic FGF, Flt-3 Ligand, Granzyme B, GROα, GROβ, IFN-α2, IL-1ra, IL-6, IL-8, IL-33, IP-10, MCP-1, MIP-1α, MIP-3α, PDGF-AA, and TRAIL. The mean values of these inflammatory factors are listed in Table 1.

### 2.2. MF-AT Inflammatory Factors and Chondrocytes/Synoviocytes Gene Expression Levels

The analysis of the influence of the number of inflammatory factors detected in the MF-AT and the therapeutic potential of this product, evaluated in terms of gene expression of OA chondrocytes and synoviocytes, showed an overall correlation, with worse results induced by more inflammatory products.

In particular, in chondrocytes, IL-8 protein release in the MF-AT was directly correlated with IL-6 gene expression. MCP-1 displayed a positive correlation with ADAMTS-5 gene level, while MIP-1α displayed a positive correlation with ADAMTS-5 gene level. Basic FGF directly correlated with IL-8 gene expression and with COX-2 gene level, while it inversely correlated with ADAMTS-4 gene level and with ADAMTS-5 gene level. More details are reported in Table 2.

In synoviocytes, Basic FGF directly correlated with HAS-1 gene expression, while it inversely correlated with TNF-α gene expression, with IL-6 gene level, with IL-8 gene expression, and with VEGF gene expression. More details are reported in Table 3.

### 2.3. Patients’ Characteristics and MF-AT Inflammatory Molecules

**Figure 1:**
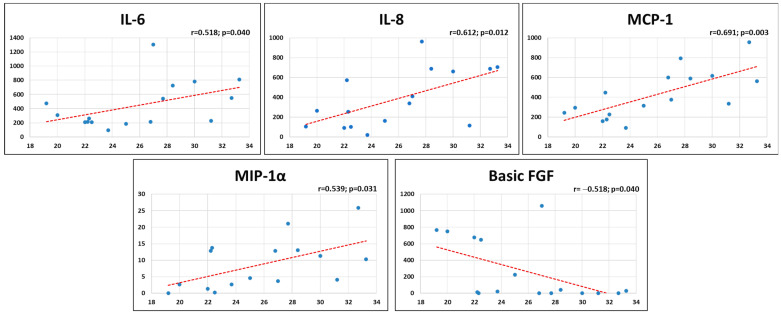
Correlations between BMI and the inflammatory factors produced by MF-AT.

**Figure 2:**
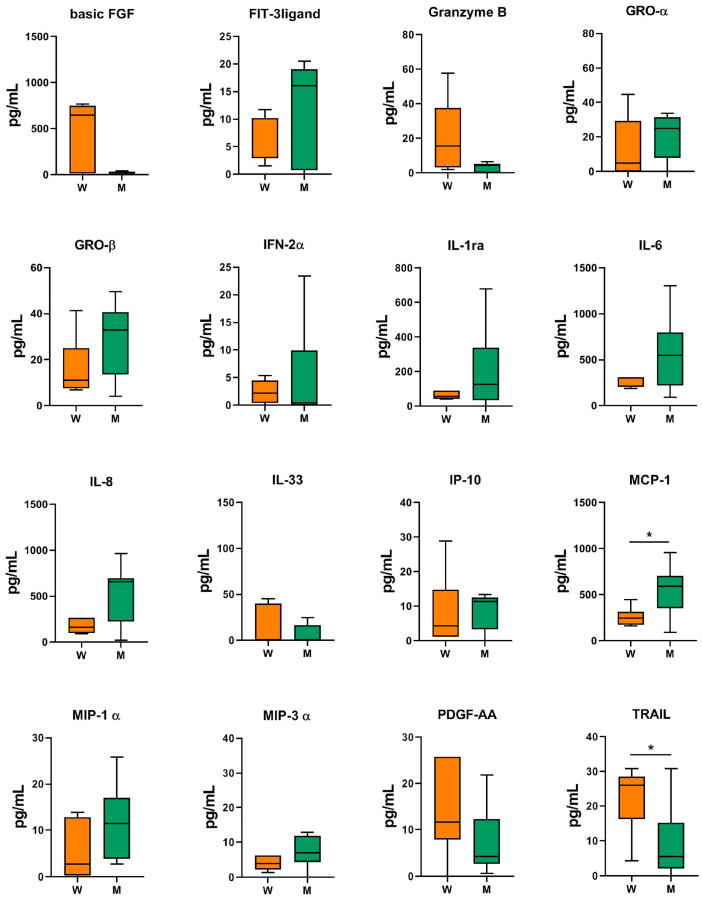
MF-AT inflammatory protein release. Data are expressed as Tukey box plots. * *p* < 0.05. W: women; M: men.

A positive correlation was found between BMI and several MF-AT inflammatory secreted factors, with a higher release of inflammatory factors in patients with higher BMI (Figure 1). In particular, BMI directly correlated with IL-6 (rho = 0.518, *p* = 0.040), IL-8 (rho = 0.612, *p* = 0.012), MCP-1 (rho = 0.691, *p* = 0.003), and MIP-1α (rho = 0.539, *p* = 0.031), while a negative correlation was found between BMI and Basic FGF (rho = −0.518, *p* = 0.040). Regarding sex, men displayed a higher release of MCP-1 with respect to women (*p* = 0.016) (Figure 2), while women had a higher expression of TRAIL (*p* = 0.045) and tended to have a higher production of Basic FGF compared to men. Age, symptoms duration, and Kellgren–Lawrence grade did not influence the release of inflammatory factors of MF-AT in this series.

### 2.4. Patients’ Characteristics and MF-AT Effects on Gene Expression

**Figure 3:**
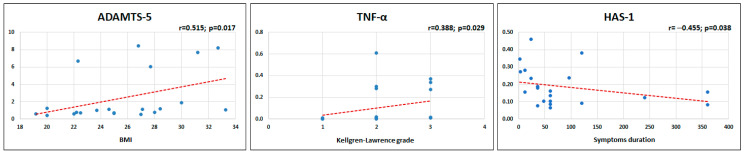
Correlations between BMI and ADAMTS-5 expression at 72 h in chondrocytes, between Kellgren–Lawrence grade and TNF-α expression at 72 h in synoviocytes, and between the symptoms duration (months) and HAS-1 expression at 72 h in synoviocytes.

**Figure 4:**
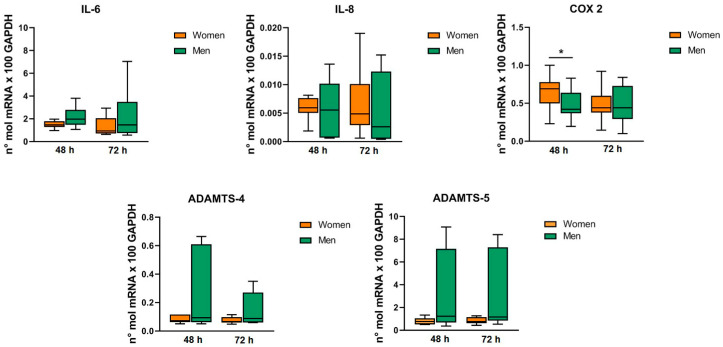
MF-AT effects on chondrocytes’ gene expression of cytokines/chemokines and catabolic enzymes. Data were normalized to GAPDH. Each graph reports data expressed as Tukey box plot. * *p* < 0.05.

**Figure 5:**
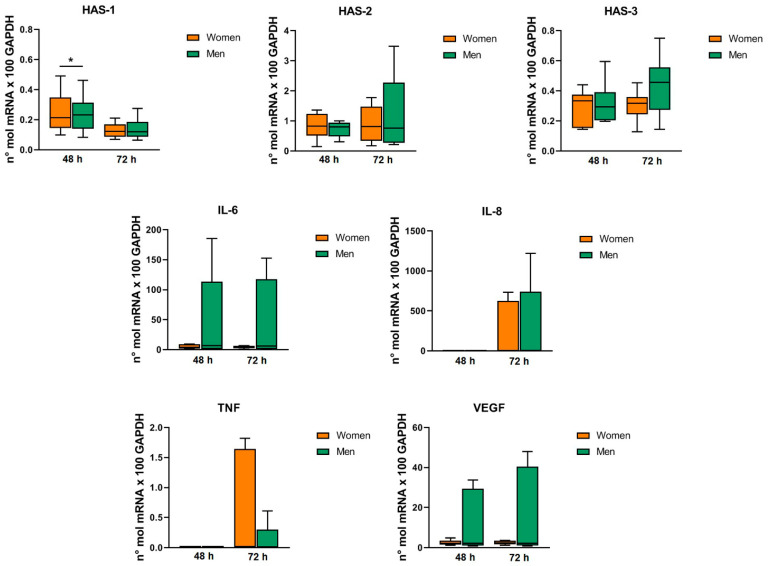
MF-AT effects on synoviocytes’ gene expression of cytokines/chemokines and extracellular matrix enzymes. Data were normalized to GAPDH. Each graph reports data expressed as a Tukey box plot. * *p* < 0.05.

BMI directly correlated with the gene expression of ADAMTS-5 in chondrocytes at 72 h (*p* = 0.017) and symptoms duration inversely correlated with the gene expression of HAS-1 in synoviocytes at 72 h (*p* = 0.038). The Kellgren–Lawrence grade directly correlated with the gene expression of TNF-α in synoviocytes at 72 h (*p* = 0.029) (Figure 3). Regarding sex (Figure 4 and Figure 5), women displayed a higher expression of COX-2 gene in chondrocytes at 48 h compared to men (*p* = 0.049), while no differences were found at 72 h. Similarly, women presented a higher expression of HAS-1 gene in synoviocytes compared to men at 48 h (*p* = 0.039), while no differences were observed at 72 h. No differences between men and women were found for the expression of the other genes in both chondrocytes and synoviocytes. Age and symptom duration did not influence the MF-AT effects on gene expression in chondrocytes and synoviocytes in this series.

## 3. Discussion

The most important finding of this study is that adipose tissue-derived products may present inflammatory properties able to influence the therapeutic potential for OA treatment, with products with a higher pro-inflammatory profile stimulating a higher expression of genes related to a more inflamed and catabolic phenotype. A higher pro-inflammatory cytokine pattern and a higher pro-inflammatory effect were found in adipose tissue-derived products obtained from OA patients with higher BMI. This suggests that overweight patients could present suboptimal properties of autologous adipose tissue-derived products used for OA treatment.

Scientific research on adipose tissue has advanced over the years. While in the past the adipose tissue was described as an inert structural organ with an energy storage function, it is now considered the largest endocrine and paracrine organ, with a potent secreting function of several adipokines and cytokines [18]. These factors can have a possible dual effect, contributing not only to its anti-inflammatory and anti-atherogenic potential, but on the other hand exerting a detrimental effect, for example in the case of obesity or metabolic syndrome [19,20]. In these circumstances, factors produced and released by adipose tissue may contribute to generating a systemic low-grade pro-inflammatory effect and a chronic inflammatory state, promoting the development of hypertension, type II diabetes mellitus, and other pathologies [21,22,23]. Beyond the systemic effects, obesity also implicates adipose tissue dysfunctions, causing adipocyte hypertrophy, increased fibrosis, impaired vascular function, and imbalanced adipokine levels, as well as the exacerbation of inflammation [24]. These dysfunctions, in particular the inflammatory component, alter the adipose tissue quality, which could alter the quality and the therapeutic potential of the obtainable adipose tissue-derived products. To verify this, this study analyzed the influence of the adipose tissue inflammatory properties on the therapeutic effects of the derived adipose product in an OA setting.

The application of MF-ATs with different inflammatory patterns in vitro to treat OA chondrocytes and synoviocytes resulted in different gene expression responses. More inflamed MF-AT produced overall worse effects compared to less inflamed products, with higher induction of pro-inflammatory or catabolic genes. In chondrocytes, positive correlations were found between IL-8 levels in MF-AT with the IL-6 mRNA expression. Within the joint, chondrocytes can increase the production of IL-6 in response to several inflammatory stimuli, which in turn may activate matrix metalloproteinases (MMPs) and ADAMTSs, contributing to cartilage degradation [25]. In chondrocytes, a positive correlation was also found between MCP-1 and MIP-1α levels in MF-AT with the mRNA expression of the ADAMTS5, which is the major aggrecanase-degrading articular cartilage matrix [26]. Controversial findings have been found regarding the influence of Basic FGF levels in MF-AT on gene expression in chondrocytes. While positive correlations were found with IL-8 and COX-2 mRNA expression, negative correlations were detected with ADAMTS4 and ADAMTS5 mRNA expression. This reflects what is already described in the literature, with inconsistent findings on the effects of this factor on OA cartilage. In fact, on one side it showed a chondroprotective role in human chondrocytes by controlling the expression and activity of the aggrecanases ADAMTS4 and ADAMTS5 [27,28], while on the other side it showed catabolic effects in human OA cartilage [29,30]. Therefore, a dual role of Basic FGF could be hypothesized, as already described in the adipose tissue, where a biphasic effect of this factor on adipogenesis has been reported [31], being involved in the negative regulation of thermogenesis of brown and beige fat [32], but at the same time, positively involved in self-renewal, differentiation, and proliferation abilities of adipose-derived MSCs [33].

An overall lower response to MF-AT based on inflammatory properties has been found in synoviocytes, with no correlations reported between inflammatory cytokines and mRNA expression. The only correlations for synoviocytes were reported for the Basic FGF levels in MF-AT. Basic FGF levels were negatively correlated to the expression of IL-6, IL-8, TNFα, and VEGF in synoviocytes, which can be related to synovial inflammation [34]. Basic FGF also positively correlated with the mRNA expression of HAS-1, an enzyme involved in the synthesis of hyaluronic acid. Deficiency of HAS-1 has been reported to result in chronic joint inflammation [35], but at the same time, its activation in response to a series of pro-inflammatory cytokines has been reported [36,37]. In OA patients, a differential expression of mRNA for the HAS isoforms has been observed, possibly in response to a differential hyaluronan metabolism [38]. In fact, although the different isoforms catalyze the same reaction, the size of their products is different [39]. In addition, in in vitro culture systems, the regulation of their expression is even more complex since it might depend on several factors such as the culture period [40], or the mechanical stimuli [41]. Overall, the response at the synovial level was more composite and complex to define, and more studies should investigate the meaning of these results.

Another important finding of this study was the influence of BMI on the inflammatory level and the therapeutic efficacy of the adipose tissue-derived products obtained from OA patients. Previous studies analyzed the profile of adipose-derived MSCs from obese patients identifying significantly different properties and potential compared to non-obese patients. In particular, samples from obese patients had an increased MSC content, but a decreased proliferative ability, an increased expression of IL-1, IL-6, and TNF-α, and a greater macrophage content [26]. Similarly, in the current study, the MF-AT obtained from patients with higher BMI showed a higher pro-inflammatory cytokine pattern and a higher pro-inflammatory effect on chondrocytes and synoviocytes. A higher presence of pro-inflammatory or catabolic molecules, such as IL-6, IL-8, MCP-1, and MIP-1α, was found in MF-AT obtained from patients with higher BMI. IL-6 expression has been already correlated with the expression of several inflammatory markers in subcutaneous adipose tissue from obese individuals [42], and adipocytes can secrete IL-6 in response to obesity, leading to an increase in adipose tissue macrophage accumulation [43]. IL-8 was found upregulated in adipocytes of obese subjects [44], and a vicious circle of IL-8 production mediated by inflammatory stimulations has been demonstrated in human adipocytes [45]. MCP-1 was found to be released at higher levels from obese individuals, and its overexpression has been considered a biomarker of an inflamed adipose organ [44,46], and together with IL-8, it can also attract monocytes/macrophages to infiltrate adipose tissue [44]. The contribution of MIP-1α to the increased infiltration of macrophages into obese adipose tissue was also described [47] and its expression was found to be elevated in white adipose tissue of obese mice and humans [48].

The role of BMI in this study is consistent with the literature on the effects of obesity. Other patients’ characteristics, however, should be considered. For example, sex differences are important as males and females differ regarding anatomical distribution in adipose tissue, sex hormone production, receptor activity, genetic influences, and gene expression [49]. Females are more protected than males against high-fat diet-induced metabolic syndrome [50], and sex also influences the production of some adipokines such as leptin and adiponectin [51]. Although the male sex has been reported as a significant risk factor for MF-AT treatment failure [52], in the current study men and women did not show overall significant differences in terms of MF-AT inflammatory pattern and MF-AT effects on chondrocytes and synoviocytes. It should be underlined that an overlap between men and patients with higher BMI was found in this study, and this is justified by the higher tendency of men toward overweight [53]. This makes the results on gender difficult to interpret, and specific gender studies are needed to understand the influence of sex on the quality and therapeutic potential of adipose-derived products for OA treatment. Regarding age, it has been reported that age affects the paracrine activity and morphology of adipose-derived MSCs [54], and an advanced age has been considered a significant risk factor for MF-AT treatment failure [52]. Nevertheless, age did not influence the release of inflammatory factors in MF-AT and its effects on chondrocytes and synoviocytes in this series. Finally, OA and obesity are strongly associated, the secretion of pro-inflammatory adipokines has been correlated with OA severity [55], and an influence of OA grade in patients’ response to MF-AT was reported [15]. However, no differences in terms of Kellgren–Lawrence OA grade were found among patients in this study. Future specific studies should investigate the influence of these patients’ characteristics on the inflammatory pattern of the adipose-derived products and their efficacy to treat knee OA.

This study presents some limitations. First, the issue of the lack of a control: cells grown in the absence of MF-AT. Nevertheless, the study’s purpose was to investigate if MF-AT showed some characteristics related to sex, BMI, and OA grade, which could affect its therapeutic effects and restrict its use in clinical practice. Thus, the comparison of a control group with the MF-AT group would provide evidence of the effects of adipose tissue on cell joints, but it would not have answered our hypothesis. Second, the number of patients analyzed did not allow further sub-analyses. Nevertheless, the number of samples evaluated is in line with previous similar studies on this topic and allowed to draw meaningful conclusions on the main study aim. Still, the protocol of the clinical study did not foresee the analysis of inflammatory markers in peripheral blood, which would have provided useful information regarding the OA-related inflammation status of patients. Then, the cells used in vitro were primary cultures isolated from OA patients, and this implies even more experimental variability. Therefore, the analysis should be extended to a larger number of cases to confirm these findings and to better investigate possible factors influencing the MF-AT quality. Finally, the amount of adipose tissue obtained from the patents available for the in vitro study was based on materials remaining from surgical procedures planned for therapeutic aims; therefore, this did not allow to deepen further the in vitro analysis. Despite a low number of analyzed patients, this study allowed to determine the influence of the inflammatory components of adipose-derived products on their effect in an OA setting and the key role of BMI as a potential influencing factor on the inflammatory properties of adipose tissue-derived products. These results could have crucial implications in the clinical practice, as the higher inflammatory pattern of adipose tissue-derived products obtained from OA patients with high BMI could reflect in a lower clinical response to this treatment. Future studies should also confirm these results in a clinical setting, as well as investigate other possible influencing factors, in order to optimize the use of autologous adipose-derived products for the injective treatment of knee OA.

## 4. Materials and Methods

### 4.1. Adipose Tissue Harvesting and Processing

Patients with knee OA scheduled to undergo an elective orthopedic procedure with adipose tissue-derived cell-based injectable therapies were enrolled. All patients signed an informed consent before the procedure and Ethics Committee approval was obtained from the Rizzoli Orthopedic Institute, Bologna, Italy (Prot. n. 0009545). A total of 21 patients (12 men and 9 women) were included, with a mean age of 51.7 ± 11.8 years and a mean BMI of 25.7 ± 4.1 kg/m^2^. The mean symptom duration was 87.1 months (range 6–360 months), while OA severity was classified based on the Kellgren–Lawrence grade as follows: 4 patients with a grade 1, 10 patients with a grade 2, and 7 patients with a grade 3. Men had a mean age of 46.6 ± 9.9 years, a mean BMI of 28.20 ± 3.09 kg/m^2^, and a mean Kellgren–Lawrence grade of 2.08 ± 0.79, whereas women had a mean age of 58.6 ± 10.9 years, a mean BMI of 22.25 ± 2.52 kg/m^2^, and a mean Kellgren–Lawrence grade of 2.22 ± 0.67. These patients underwent selective liposuction from the subcutaneous abdominal fat. Before fat harvesting, the site was injected with a saline solution with adrenaline and lidocaine (500 mL), then adipose tissue was collected using a 13-gauge blunt cannula connected to a 20-mL Vaclock syringe. The harvested fat was immediately processed using the Lipogems system (Lipogems International Spa, Milan, Italy), a system that uses mild mechanical forces in a completely closed system, avoiding enzymes, additives, and other manipulations. The size of adipose tissue clusters was progressively reduced, while oily substances, cell debris, and blood residue were eliminated to obtain a micro-fragmented adipose tissue (MF-AT). This product encompasses a preserved vascular stroma with slit-like capillaries wedged between adipocytes and stromal stalks containing vascular channels and cells with pericyte/MSC identity.

After fat processing, a small fraction of residual MF-AT was sent to the laboratory of the same institute for in vitro analyses.

### 4.2. Release of Inflammatory Factors from Adipose Tissue

The analysis of the MF-AT supernatant was performed to analyze the release of inflammatory factors from the product to determine its inflammatory component. Supernatants from MF-AT were collected centrifuging 0.2 mL of MF-AT for 10 min at 1500 rpm and stored at −80 °C until use, without any culture period. Successively, samples were evaluated for the release of CD40 Ligand, EGF, Eotaxin, FGF basic, Flt-3 Ligand, G-CSF, GM-CSF, Granzyme B, GROα, GROβ, IFN-α2, IFN-β, IFN-γ, IL-1α, IL-1β, IL-1ra, IL-2, IL-3, IL-4, IL-5, IL-6, IL-7, IL-8, IL-9, IL-10, IL-12p70, IL-13, IL-15, IL-17A, IL-17E, IL-33, IP-10, MCP-1, MIP-1α, MIP-1β, MIP-3α, MIP-3β, PDGF-AA, PDGF-AA/BB, PD-L1/B7-H1, RANTES, TGF-α, TNF-α, TNF-β, TRAIL, and VEGF using Human XL Cytokine Luminex^®^ Performance Assay 46-plex Fixed Panel (Bio-techne, R&Dsystems, Minneapolis, MN, USA), according to the manufacturer’s protocol.

The influence of the patients’ demographic characteristics on the inflammatory properties of MF-AT was analyzed for sex, age, BMI, symptoms duration, and Kellgren–Lawrence grade.

### 4.3. Chondrocyte and Synoviocyte Isolation and Co-Colture Experiments

Co-colture experiments on chondrocytes and synoviocytes were performed to evaluate the therapeutic potential of the MF-AT based on its inflammatory properties. Chondrocytes (1 man and 2 women, aged 67 ± 12 years) and synoviocytes (1 man and 5 women, aged 67 ± 7 years) were isolated from the knees of OA patients who underwent joint replacement surgery. Cartilage was removed from the femoral condyle and minced into small pieces. Chondrocytes were isolated by sequential enzymatic digestions: 1 h with pronase (Sigma-Aldrich, St. Louis, MO, USA) and 1–2 h with 0.2% collagenase (Sigma-Aldrich) at 37 °C. Successively, isolated cells were filtered by nylon meshes (100 μm and 70 μm, respectively), washed, and centrifuged. Chondrocytes were seeded at a concentration of 8 × 10^3^ cells/cm^2^ in T150 flasks and cultured under conventional monolayer culture conditions in DMEM (Sigma-Aldrich) with 10% FCS. Once reached confluency, chondrocytes from each patient were frozen at passage 0 and stored until use. Synoviocytes were isolated by mincing synovium into small pieces and cultured in Optimem (Gibco-BRL, Life Technologies, Grand Island, NY, USA) culture medium supplemented with 10% FCS for two weeks in order to allow the release of the cells. Synoviocytes from each patient were frozen at passage 0 and stored until use. When enough cell samples were collected, chondrocytes and synoviocytes were thawed and seeded at 8 × 10^3^ cells/cm^2^ in T150 flasks; at confluence, they were pooled and used for the experiments. For chondrocytes and synoviocytes, three different experiments were performed, and for each experiment, the same batch of pooled cells were used.

The effects of MF-AT were evaluated on both pooled chondrocytes and synoviocytes in a co-culture system. Briefly, co-cultures were set up by seeding 1.5 × 10^5^/cm^2^ chondrocytes/synoviocytes in the lower chamber of a 24-well plate and 0.2 mL of MF-AT in the transwell (0.4 μm pore size, Corning, Toledo, OH, USA) in DMEM (Sigma-Aldrich) with 10% FCS and all evaluations were performed at 48 and 72 h. Chondrocytes and synoviocytes were used at passage 2/3.

### 4.4. MF-AT Effects on Gene Expression in Chondrocytes and Synoviocytes

Cells from co-cultures previously treated with MF-AT were analyzed by Real-Time RT-PCR at 48 and 72 h to investigate the expression of several genes involved in OA pathophysiology, including inflammatory cytokines/chemokines (IL-6, IL-8, and TNF-α), extracellular matrix enzymes (HAS-1, HAS-2, HAS-3, ADAMTS-4, and ADAMTS-5), and molecules related to angiogenesis/pain (VEGF and COX-2) (primer sequences are listed in Table 4).

Total RNA was isolated using TRIZOL reagent (Invitrogen, Thermo Fisher Scientific Inc., Waltham, MA, USA) following the manufacturer’s protocol. Successively, RNA samples were treated with DNase I (DNA-free Kit; Ambion, Life Technologies, Austin, TX, USA) and quantified by Nanodrop spectrophotometer (Euroclone, Pero, Italy). The RNA was reverse transcribed using the SuperScript Vilo cDNA synthesis Kit (Invitrogen, Carlsbad, CA, USA), according to the manufacturer’s recommended protocol. Real-Time PCR was performed in a LightCycler Instrument (Roche Molecular Biochemicals, Indianapolis, IN, USA) using SYBR Premix Ex Taq (Takara, Clontech Laboratories, Mountain View, CA, USA) with the following protocol: initial activation at 95 °C for 10 min, amplification for 45 cycles at 95 °C for 5 s and at 60 °C for 20 s. mRNA levels for each target gene were calculated and normalized using the reference gene GAPDH according to the DDCt method. Data were expressed as “Number of molecules per 100 GAPDH”.

The influence of the patients’ demographic characteristics on the gene expression after MF-AT stimulation was analyzed for sex, age, BMI, symptoms duration, and Kellgren–Lawrence grade.

### 4.5. Statistical Analysis

Repeated measures GLM with post-hoc Sidak correction for multiple comparisons was performed to compare the gene expression at different follow-up times. The Mann–Whitney non-parametric test evaluated by exact method was performed to assess the between groups differences. The Spearman rank correlation was used to assess correlations between continuous data. For all tests, *p* < 0.05 was considered significant. All statistical analyses were performed using SPSS v.19.0 (IBM Corp., Armonk, NY, USA).

## 5. Conclusions

This study demonstrated that adipose tissue-derived products may present inflammatory properties able to influence the therapeutic potential for OA treatment, with products with a higher pro-inflammatory profile stimulating a higher expression of genes related to a more inflamed and catabolic phenotype. A higher pro-inflammatory cytokine pattern and a higher pro-inflammatory effect were found in adipose tissue-derived products obtained from OA patients with higher BMI. Patients’ characteristics should be considered when planning the use of autologous adipose-derived products for the injective treatment of OA.

## Figures and Tables

**Table 1:** Inflammatory factors detected in the MF-AT supernatants.

Inflammatory Factors	Mean (pg/mL)	SD
Basic FGF	264.1	372.6
Flt-3 Ligand	9.1	7.8
Granzyme B	12.6	17.1
GROα	16.7	19.7
GROβ	23.0	15.1
IFN-α2	4.1	6.4
IL-1ra	149.9	175.4
IL-6	444.1	326.2
IL-8	383.2	290.5
IL-33	15.5	27.9
IP-10	9.9	9.4
MCP-1	424.2	243.2
MIP-1α	8.8	7.6
MIP-3α	7.4	7.6
PDGF-AA	10.9	8.8
TRAIL	14.7	11.3

**Table 2:** MF-AT inflammatory factors correlation with chondrocytes’ gene expression.

Inflammatory Factors	Gene Expression (48 h)	Gene Expression (72 h)
IL-8	IL-6: rho = 0.591; *p* = 0.016	IL-6: rho = 0.574; *p* = 0.020
MCP-1	None	ADAMTS-5: rho = 0.547; *p* = 0.028
MIP-1α	ADAMTS-5: rho = 0.736; *p* = 0.001	ADAMTS-5: rho = 0.745; *p* = 0.001
Basic FGF	IL-8: rho = 0.630; *p* = 0.009 COX-2: rho = 0.876; *p* = 0.0008 ADAMTS-4: rho = −0.603; *p* = 0.013 ADAMTS-5: rho = −0.959; *p* < 0.0005	IL-8: rho = 0.524; *p* = 0.030 COX-2: rho = 0.536; *p* = 0.030 ADAMTS-4: rho = −0.638; *p* = 0.008 ADAMTS-5: rho = −0.929; *p* < 0.0005

**Table 3:** MF-AT inflammatory factors correlation with synoviocytes’ gene expression.

Inflammatory Factors	Gene Expression (48 h)	Gene Expression (72 h)
Basic FGF	HAS-1: rho = 0.844; *p* < 0.0005 TNF-α: rho = −0.624; *p* = 0.010 IL-6: rho = −0.494; *p* = 0.052 IL-8: rho = −0.515; *p* = 0.041 VEGF: rho = −0.521; *p* = 0.039	HAS-1: rho = 0.807; *p* < 0.0005 TNF-α: rho = −0.805; *p* < 0.0005 IL-6: rho = −0.509; *p* = 0.044 IL-8: rho = −0.569; *p* = 0.021 VEGF: rho = −0.639; *p* = 0.008

**Table 4:** List of primers used in Real-Time PCR.

RNA Template	Primer Sequences (5′-3′)	Annealing Temperature (°C)
GAPDH	5′-TGGTATCGTGGAAGGACTCATGAC 3′-ATGCCAGTGAGCTTCCCGTTCAGC	60
IL-6	5′-TAGTGAGGAACAAGCCAGAG 3′-GCGCAGAATGAGATGAGTTG	60
IL-8	5′-CCAAACCTTTCCACCC 3′-ACTTCTCCACAACCCT	60
TNF-α	5′-AGCCCATGTTGTAGCAAACC 3′-ACCTGGGAGTAGATGAGGTA	60
COX-2	5′-CAGCACTTCACGCATCAGTTT 3′-GCGCAGTTTACGCTGTCTA	60
HAS-1	5′-TGGTGCTTCTCTCGCTCTACG 3′-GAACTTGGCAGGCAGGAGG	60
HAS-2	5′-AAATGGGATGAATTCTTTGTTTATG 3′-GGCGGATGCACAGTAAGGAA	60
HAS-3	5′-CAGCTGATCCAGGCAATCGT 3′-TGGCTGACCGGATTTCCTC	60
VEGF	5′-TGATGATTCTGCCCTCCTC 3′-GCCTTGCCTTGCTGCTC	60
ADAMTS-4	5′-CTGCCTACAACCACCG 3′-GCAACCAGAACCGTCC	60
ADAMTS-5	5′-GCACTTCAGCCACCATCAC 3′-AGGCGAGCACAGACATCC	60

## Data Availability

Not applicable.

## Abbreviations

ADAMTS	a disintegrin and metalloproteinase with thrombospondin motifs
BMI	body mass index
CD	cluster of differentiation
COX	cyclooxygenase
DMEM	Dulbecco’s modified eagle medium
EGF	epidermal growth factor
FGF	fibroblast growth factor
Flt-3 ligand	Fms-like tyrosine kinase 3 ligand
GAPDH	glyceraldehyde-3-phosphate dehydrogenase
G-CSF	granulocyte colony-stimulating factor
GM-CSF	granulocyte-macrophage colony-stimulating factor
GRO	growth-related oncogene
HAS	hyaluronan synthase
IFN-α2	interferon alpha-2
IL	interleukin
IL-1ra	interleukin-1 receptor antagonist
MCP-1	monocyte chemoattractant protein-1
MF-AT	micro-fragmented adipose tissue
MIP	macrophage inflammatory protein
MMPs	matrix metalloproteinases
mRNA	messenger ribonucleic acid
MSCs	mesenchymal stromal cells
OA	osteoarthritis
PD-L1	programmed death-ligand 1
PDGF	platelet-derived growth factor
RT-PCR	reverse transcriptase-polymerase chain reaction
TGF	transforming growth factor
TNF	tumor necrosis factor
TRAIL	tumor necrosis factor-related apoptosis-inducing ligand
VEGF	vascular endothelial growth factor
